# Isolated dysphagia masking myasthenia gravis diagnosis

**DOI:** 10.1055/s-0046-1817045

**Published:** 2026-03-11

**Authors:** Isabella Lopes Lusvarghi, Eduarda Heringer Bernis, Rafaela Gatti Lopes, Rafaela Costa Vieira, Breno Franco Silveira Fernandes, Davi Teixeira Urzêdo Queiroz

**Affiliations:** 1 Hospital Felício Rocho, Departamento de Gastroenterologia e Hepatologia, Belo Horizonte MG, Brazil.; 2 Hospital Felício Rocho, Departamento de Neurologia e Neurocirurgia, Belo Horizonte MG, Brazil.

**Keywords:** Myasthenia Gravis, Deglutition Disorders, Thymoma, Neuromuscular Junction

## Abstract

Myasthenia gravis (MG) is an autoimmune disorder of the neuromuscular junction characterized by fluctuating skeletal muscle weakness. We report a 61-year-old woman presenting with progressive dysphagia as the sole initial symptom for 15 months, leading to diagnostic delay. Esophageal manometry suggested ineffective motility, and electroneuromyography was normal, reinforcing a non-neurological hypothesis. During hospitalization for worsening dysphagia, cervical magnetic resonance imaging (MRI) incidentally revealed an anterior mediastinal nodule consistent with thymoma. Pulse corticosteroid therapy caused abrupt clinical deterioration, prompting neurological evaluation. Pyridostigmine produced marked improvement within 48 hours, and acetylcholine receptor antibody testing confirmed MG (18.7 nmol/L, radioimmunoassay). Robotic thymectomy revealed a type AB thymoma. The patient remains stable on azathioprine 2.5 mg/kg/day, with oncologic follow-up. This case highlights that MG should be considered in persistent unexplained dysphagia, particularly when symptoms worsen after corticosteroid exposure. Early recognition avoids morbidity and unnecessary invasive procedures.

## CLINICAL VIGNETTE

A 61-year-old woman, a retired hairdresser from a rural area in the state of Minas Gerais, Brazil, presented with a 15-month history of progressive oropharyngeal dysphagia. The symptoms began insidiously and were initially characterized by difficulty swallowing solid and pasty foods. Over time, she developed sialorrhea, nasal regurgitation, and significant weight loss. She denied dysphonia, diplopia, ptosis, limb weakness, or sensory symptoms. The dysphagia was initially more pronounced in the evening, suggesting fatigability, but gradually became constant throughout the day.

Initial evaluation focused on a gastroenterological etiology. Esophageal manometry demonstrated ineffective esophageal motility, leading to the hypothesis of a primary motility disorder. A videofluoroscopic swallowing study revealed impaired oral propulsion, moderate to severe oropharyngeal residue, and laryngeal penetration and aspiration with liquids (PAS 7), compatible with severe oropharyngeal dysphagia. Electroneuromyography revealed normal motor and sensory conduction studies and normal needle electromyography (EMG) of the genioglossus muscles, without evidence of denervation or neuromuscular-junction dysfunction. No significant decrement (< 5%) was observed on repetitive nerve stimulation at 3 Hz in the facial and trapezius muscles, and single-fiber EMG was not performed. These normal findings reinforced the initial assumption of a non-neurological cause.

A comprehensive rheumatological panel, including creatine kinase, antinuclear antibodies (ANA), anti-SSA, anti-SSB, and other myositis-specific antibodies, returned negative. Thyroid function and autoimmune screening were unremarkable, further reducing the likelihood of systemic autoimmune or inflammatory myopathies.

**Figure 1:**
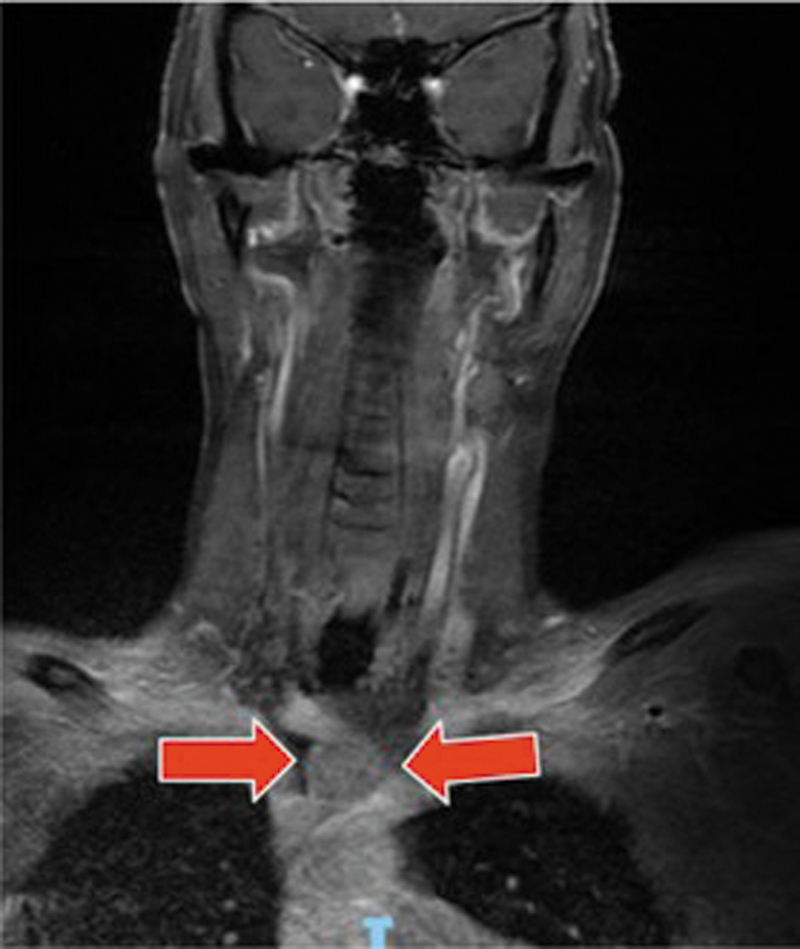
Coronal T1-weighted contrast-enhanced MRI demonstrating a well-defined nodular lesion in the anterior mediastinum (red arrows), consistent with thymoma.

Due to significant worsening of dysphagia and nutritional compromise, the patient was admitted for inpatient evaluation. During hospitalization, cervical magnetic resonance imaging (MRI) incidentally detected an anterior mediastinal nodule ( Figure 1 ), consistent with thymic tissue. The patient underwent pulse corticosteroid therapy (methylprednisolone 1 g/day for 3 days) to treat a presumed inflammatory myopathy, but she developed abrupt worsening of swallowing function and new-onset facial weakness shortly thereafter.

Following this deterioration, neurology was consulted. Clinical examination revealed mild bilateral facial weakness, nasal speech, and fatigable dysarthria, without limb or ocular involvement. Given the clinical context, pyridostigmine (60 mg every 6 hours) was initiated, resulting in marked improvement of swallowing, salivary control, phonation, and facial weakness within 48 hours.

Serum acetylcholine receptor (AChR) antibodies were positive at 18.7 nmol/L (radioimmunoassay), confirming the diagnosis of generalized MG. Chest computed tomography (CT) subsequently confirmed a mediastinal mass consistent with thymoma.

**Figure 2:**
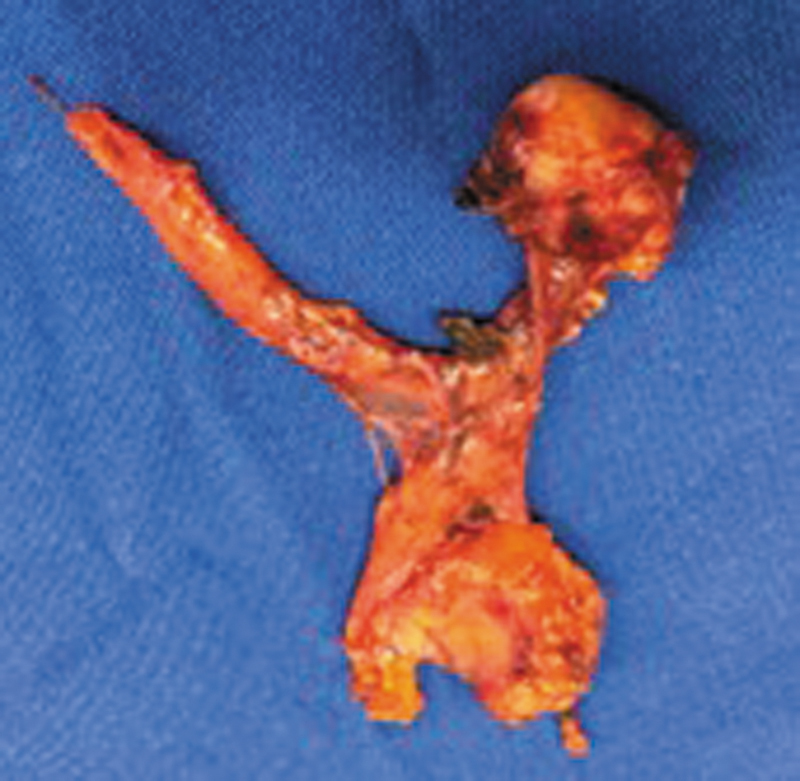
Surgical specimen following robotic thymectomy, showing a well-demarcated thymic mass with lobulated contours and adjacent thymic tissue, consistent with thymoma.

The patient underwent robotic thymectomy ( Figure 2 ). Histopathological examination confirmed a thymoma, and immunohistochemical analysis supported a type-AB thymoma, characterized by epithelial neoplastic cells positive for AE1/AE3, p63, and CK5/6, and scattered lymphocytes positive for TdT. The tumor exhibited a low proliferative index (Ki-67 < 5%) and absence of neuroendocrine marker expression, consistent with a thymoma with malignant potential.

In the immediate postoperative period, the patient experienced substantial clinical recovery, allowing progressive reintroduction of oral feeding and removal of the gastrostomy tube.

At one-month follow-up, she had resumed a regular oral diet, regained weight, and was maintained on azathioprine 2.5 mg/kg/day for immunosuppressive therapy. Positron emission tomography – CT (PET-CT) showed no evidence of metastatic disease, but adjuvant radiotherapy was planned due to histopathological features suggestive of malignancy. Systemic chemotherapy was not indicated.

She remains clinically stable under combined neurological and oncological follow-up for thymoma surveillance.

## FROM PRESENTATION TO RESOLUTION: LESSONS LEARNED

### What makes oropharyngeal dysphagia a diagnostic challenge?

Oropharyngeal dysphagia represents a clinical interface between neurology and gastroenterology, and its etiological spectrum encompasses structural, inflammatory, and neuromuscular disorders. Identifying the underlying cause is challenging because the swallowing mechanism involves multiple muscular and neural components, any of which can be affected at different levels of the neuraxis or neuromuscular junction. 1 2 3

In MG, bulbar and oropharyngeal weakness may appear at disease onset or as part of a generalized presentation, but isolated dysphagia as the initial and sole manifestation is rare, accounting for less than 6% of cases. 2 This rarity often delays diagnosis and misleads clinicians toward primary esophageal or otorhinolaryngologic causes. 3 The fluctuation and fatigability of symptoms—hallmarks of MG—may initially be subtle and overlooked when dysphagia becomes persistent rather than intermittent, as observed in our patient.

Another diagnostic pitfall is the potential overlap between achalasia and neuromuscular junction disorders. Achalasia has been reported in association with thymoma and MG, likely reflecting autoimmune cross-reactivity within the enteric nervous system. 4 5 6 In such cases, esophageal manometry may reveal nonspecific motility disturbances similar to those found in our patient, underscoring the importance of correlating gastroenterological findings with a detailed neuromuscular assessment.

Additionally, electrophysiological studies may yield normal results early in the disease course, particularly when testing proximal or cranial muscles. Repetitive nerve stimulation (RNS) in limb or facial muscles may not capture bulbar involvement, while single-fiber EMG—the most sensitive diagnostic tool—was not initially performed in this case. 7 Therefore, clinicians must interpret normal electrodiagnostic findings cautiously, especially in the presence of fluctuating or unexplained bulbar symptoms.

### Why was the diagnosis initially missed in this case? What role did corticosteroids play?

The diagnostic delay in this patient illustrates how specialty-specific anchoring can mislead the investigation of dysphagia. Because the manometric findings suggested esophageal dysfunction, the initial work-up remained restricted to the gastroenterological domain. Moreover, the absence of ocular, limb, or respiratory weakness made a neuromuscular cause seem unlikely, which was further reinforced by a normal electroneuromyography.

However, the turning point occurred when pulse corticosteroid therapy was administered for presumed inflammatory myopathy, resulting in abrupt clinical worsening. Corticosteroids can transiently exacerbate MG, particularly when high doses are introduced rapidly. 8 9 This paradoxical response occurs in up to 50% of untreated patients and is thought to reflect enhanced postsynaptic receptor blockade and temporary immune activation before clinical improvement sets in. The steroid-induced deterioration in this case was a key clinical clue that prompted neurological reevaluation. 9

Furthermore, thymoma-associated MG, as in this patient, represents a distinct immunopathological subtype characterized by aberrant thymic epithelial expression of acetylcholine receptor (AChR) epitopes and peripheral autoantibody production. 10 These patients often exhibit more severe bulbar involvement and poorer initial response to corticosteroids alone, emphasizing the importance of early recognition and surgical management.

### How can misdiagnosis be avoided in similar cases?

This case emphasizes several practical points for clinicians confronting unexplained dysphagia, particularly when the clinical presentation is isolated and insidious.

First, it is essential to maintain a broad differential diagnosis that includes neurogenic, myopathic, and neuromuscular junction causes. Some neuromuscular conditions associated with oropharyngeal dysphagia are summarized below.

Motor neuron disease (progressive bulbar palsy, amyotrophic lateral sclerosis) should always be considered in chronic, progressive dysphagia, particularly in older adults. 11 However, unlike in MG, these patients typically present with mixed upper and lower motor neuron signs, including spastic dysarthria, tongue fasciculations, and brisk jaw reflexes, as well as progressive weakness without fluctuation or diurnal variation. In our patient, the absence of tongue atrophy, fasciculations, or upper motor neuron signs, combined with a rapid response to pyridostigmine, effectively ruled out motor neuron disease.

Inflammatory myopathies, such as polymyositis, dermatomyositis, and autoimmune necrotizing myopathy, often cause pharyngeal weakness and dysphagia, but they typically present with elevated creatine kinase levels and proximal limb weakness. 12 In the present case, the normal CK and negative myositis antibody panel made this etiology unlikely.

Inclusion body myositis (IBM) is another important differential, especially in elderly patients with slowly progressive dysphagia. However, IBM is distinguished by asymmetric quadriceps and finger flexor weakness, poor response to immunotherapy, and characteristic muscle biopsy findings, 12 none of which were observed here.

Hereditary myopathies, particularly oculopharyngeal muscular dystrophy (OPMD), may also manifest with dysphagia, but these conditions are generally accompanied by ptosis, ophthalmoparesis, or myotonia. Often, a positive family history can be found. 13 Our patient's late-onset, isolated presentation, and lack of family history excluded these possibilities.

Finally, structural or esophageal causes, including achalasia, must be differentiated from neurogenic dysphagia. Achalasia can rarely coexist with MG and thymoma, as reported in the literature, 4 5 6 and was initially suspected in this case due to manometric abnormalities. However, the later appearance of fatigability, steroid-induced worsening, and antibody positivity clarified the diagnosis.

In addition to these diagnostic considerations, clinicians should remain vigilant for contextual clues suggestive of MG, such as fluctuating symptoms, involvement of multiple bulbar functions (speech, swallowing, facial expression), and paradoxical worsening after corticosteroid use. 8 9

A multidisciplinary approach—involving neurologists, gastroenterologists, and speech-language pathologists—remains the cornerstone for accurate diagnosis and management of complex dysphagia cases. Early collaboration can prevent diagnostic delay, unnecessary invasive procedures, and treatment-related complications.

## Notes

TEACHING POINTS

1. Myasthenia gravis (MG) may rarely present with isolated dysphagia, which can mimic gastroenterological disorders and delay diagnosis.
2. Symptom worsening after corticosteroid therapy and improvement with pyridostigmine are important diagnostic clues.
3. When faced with unexplained oropharyngeal or esophageal dysphagia, clinicians should include neuromuscular junction disorders in the differential diagnosis.
